# The science of safety: adverse effects of GLP-1 receptor agonists as glucose-lowering and obesity medications

**DOI:** 10.1172/JCI194740

**Published:** 2026-02-16

**Authors:** Ryan J. Jalleh, Nicholas J. Talley, Michael Horowitz, Michael A. Nauck

**Affiliations:** 1 Endocrine and Metabolic Unit, Royal Adelaide Hospital, Adelaide, Australia.; 2 Adelaide Medical School, The University of Adelaide, Adelaide, Australia.; 3 School of Medicine and Public Health, College of Health, Medicine and Wellbeing, University of Newcastle, Callaghan, New South Wales, Australia.; 4 Diabetes, Endocrinology, Metabolism Section, Medical Department I, Katholisches Klinikum Bochum gGmbH, Sankt Josef-Hospital, Ruhr-University, Bochum, Germany.; 5 Institute for Clinical Chemistry and Laboratory Medicine, University Medicine Greifswald, Greifswald, Germany.

## Abstract

GLP-1 receptor agonist (GLP-1RA) medications have transformed the treatment of type 2 diabetes (T2D) and obesity, with robust evidence for cardiovascular and renal benefits. Nevertheless, GLP-1RA therapy is associated with a pattern of adverse events affecting their safety and tolerability. Here, we delineate mechanisms potentially leading to adverse responses to GLP-1RAs, describe the impact of side effects on treatment persistence, discuss potential mitigation strategies, and identify areas requiring further studies. Concerns that GLP-1RAs raise the risk for acute pancreatitis and pancreatic cancer have been dispelled by long-term clinical trials. However, GLP-1RAs may confer an increased risk for thyroid cancer. Sight-threatening eye complications resulting from rapid reductions in glycemia may be avoided by retinal screening and ophthalmologic treatment before GLP-1RA initiation. The slowing of gastric emptying with GLP-1RA treatment increases the propensity for retained gastric contents, which could increase the risk of aspiration during upper gastrointestinal endoscopy or general anesthesia. These risks may, however, be elevated in individuals with long-standing T2D even in the absence of GLP-1RA treatment. Improved pharmacovigilance and a more standardized, quantitative assessment of adverse events in clinical trials, particularly in the assessment of gastrointestinal symptoms, would facilitate definition of the benefit-risk relationship for individual medications and indications.

Among the two incretin hormones, glucose-dependent insulinotropic polypeptide (GIP) and glucagon-like peptide-1 (GLP-1), the latter has been established as having therapeutic potential for reducing elevated plasma glucose (1, 2) and body weight (3), while the former has been unable to either stimulate insulin secretion (1) or lower plasma glucose effectively in type 2 diabetes (T2D) (4). Therefore, GLP-1 became the parent compound for the development of GLP-1 receptor agonists (GLP-1RAs) (5–7). GLP-1 acts through a class 2 member of the G-coupled protein C receptor family seven-transmembrane receptor (GLP-1R) (8), which is mainly expressed in pancreatic islets, the brain, and the gastrointestinal (GI) tract (9–12), and less prominently in the kidney and eyes (11–13). This class of drugs is now used widely, with beneficial GLP-1R–mediated effects on body weight and glycemic control, as well as their cardio-renal complications (7, 14). The stimulation of GLP-1R, however, may also elicit adverse events. The focus of this Review is on adverse events associated with the treatment with selective GLP-1RAs, or with the dual-targeted GIP receptor agonist (GIPRA)/GLP-1RA tirzepatide, the overall treatment uptake, adherence, and persistence of which have been suboptimal. We discuss adverse events of specific interest observed with these therapies (and their potential underlying mechanisms), including GI side effects, psychiatric, ocular, thyroid, biliary, and pancreatic complications (Table 1).

## GI side effects

A recent systematic review of 39 randomized controlled trials of GLP-1RAs showed a class effect of increased risks of nausea, vomiting, diarrhea and constipation compared with placebo (15) in individuals without diabetes. For the newer-generation therapies there was increased risk of nausea compared with placebo: semaglutide relative risk (RR) 2.95 (95% CI 2.61–3.32), tirzepatide RR 2.90 (95% CI 2–4.19), and orforglipron RR 4.77 (95% CI 2.02–11.31). In another systematic review that included individuals with T2D from 38 phase III or IV placebo-controlled randomized controlled trials with GLP-1RA–based therapy (16), nausea was reported in 19.3% of participants with active treatment versus 6.5% with placebo, and vomiting was reported in 7.6% of participants versus 2% with placebo. The odds ratios (vs. placebo treatment) for nausea and vomiting were similar for various compounds/preparations of GLP-1RA–based medications, while intended effect sizes (glycemic control and body weight reduction) varied widely (Table 2). More elaborate dose escalation regimens were associated with greater efficacy,

GI adverse events not infrequently lead to discontinuation of drug treatment, both in randomized clinical trials (16, 17) and in real-world practice (18, 19). In a systematic review of randomized clinical trials, 6.5% of those using a GLP-1RA discontinued it due to adverse events versus 3.6% of those on placebo (16), but these rates appear to be higher in observational studies where factors associated with discontinuation were an age of 65 years or older and GI adverse events with treatment (21). In the longer-term trials that reported individual adverse event categories associated with discontinuation of drug treatment, nausea was shown to be the leading cause of discontinuation, followed by vomiting and diarrhea, with much lower risks associated with constipation, abdominal discomfort, or pain (Table 3). It is possible that optimizing dose escalation schedules will improve tolerability, and this should be further explored in future studies (20). In a recent phase II trial with subcutaneous semaglutide, when doses were escalated relatively rapidly to 16 mg weekly, participants experienced additional weight loss, but at the expense of more adverse effects (21). However, it should be appreciated that a substantial proportion (>50%) of the participants did not report any GI adverse events, indicating that those individuals may be able to tolerate even higher doses. More individualized approaches to dose escalation may be needed to maximize the therapeutic potential for those who tolerate GLP-1RAs well, and to minimize the frequency and severity of adverse events in those who are more susceptible.

*Does GIPR agonism improve GLP-1RA tolerance?* Tirzepatide, a coagonist of GIPR and GLP-1R, is characterized by greater efficacy in both glucose lowering and weight loss than can be achieved with current selective GLP-1RAs (16), while having a comparable adverse event profile. In animal experiments involving shrews (which are able to vomit), GLP-1RAs elicit frequent episodes of vomiting, while GIPRAs do not. Moreover, the GIPRA GIP-085 almost totally prevented vomiting induced by a long-acting GLP-1RA GLP-140 (22). In an exploratory clinical trial, a long-acting GIPRA tended to reduce GI adverse events in response to rapidly escalated liraglutide in healthy individuals (23). In the SURPASS-2 trial, tirzepatide at doses of 5, 10, and 15 mg/week were compared with 1 mg/week semaglutide, and the 5 mg/week tirzepatide dose was found to be only slightly more effective than semaglutide in reducing both HbA_1c_ (–2.01% vs. –1.86%) and body weight (–7.6 vs. –5.7 kg). Under these conditions, overall GI adverse events were, however, only slightly less prevalent with tirzepatide (40.0% vs. 43.0%), with minor differences in favor of tirzepatide for nausea (17.4% vs. 19.2%) and vomiting (5.7% vs. 8.1%), and, less so, diarrhea (13.2% vs. 13.7%) (24). In the systematic review of clinical trials mentioned above, tirzepatide conferred the greatest risk of vomiting, RR 13.23 (95% CI 4.85–36.09), whereas the risks for semaglutide (RR 4.21 [95% CI 3.58–4.95]) and orforglipron (RR 4.43 [95% CI 1.45–13.56]) were similar (15). Moreover, in a recent large cardiovascular outcomes trial (SURPASS-CVOT), numerically higher proportions of participants taking tirzepatide (15 mg weekly) reported nausea, vomiting, and diarrhea compared with those taking the selective GLP-1RA dulaglutide (1.5 mg weekly) (25). Although comparisons between tirzepatide and higher doses of dulaglutide (e.g., 4.5 mg) have yet to be done, these data argue against the concept that GIP coagonism can reduce the risk of GI adverse events. With the advent of dual and triple agonists stimulating additional receptors of gastro-entero-pancreatic peptide hormones with therapeutic potential, there is the possibility of altered adverse event profiles. Table 4 summarizes potential effects of combined stimulation of receptors for GIP, glucagon, amylin, and peptide YY on adverse events.

### Assessment of GI symptoms.

In the vast majority of trials, symptoms are evaluated by participant “self-report” that, while simple, has major limitations (26). Firstly, in the absence of a standardized questionnaire providing precise definitions of terms describing individual adverse effects, symptoms are likely to be perceived differently by participants. For example, uncomfortable fullness has the potential to be reported as nausea by one participant, but not another. Therefore, precise definitions, in terms understandable to laypeople, are required. Secondly, with self-reporting of symptoms there may be the expectation of adverse GI side effects (i.e., the nocebo effect). Thirdly, GI symptoms are common in healthy adults, with a greater frequency in individuals with diabetes even if they are not using a GLP-1RA, and these symptoms characteristically fluctuate (27). Therefore, changes in GI symptoms should also be quantified, rather than simply assessing their presence or absence. The ideal subject-reported outcome instrument would employ precise definitions for each symptom and their degree of severity, provide reliable inter-interviewer reproducibility, and be sensitive to the detection of changes in symptoms following initiation of the drug and any subsequent dose changes. In the assessment of treatment outcomes in functional GI disorders such as irritable bowel syndrome (28), the US Food and Drug Administration (FDA) and European Medicine Agency (EMA) have mandated the use of validated questionnaires. We believe that it would be beneficial for a validated instrument to be developed for the evaluation of GI adverse effects in GLP-1RA trials. Table 3 and Supplemental Table 1 represent preliminary examples of the proposed content for such instruments. In the interim, it should be appreciated that several instruments have been used widely in the evaluation of symptoms associated with GI disorders, including the Bowel Disease Questionnaire (BDQ) (29), a comprehensive questionnaire with 71 precisely defined questions. Derived from the BDQ is the Diabetes Bowel Symptom Questionnaire, which also contains items specific for diabetes and its complications (30). Both of these questionnaires, are, however, time-consuming for the participant to complete. Abbreviated instruments include the patient assessment of upper GI symptom severity index (PAGI-SYM) (31), the diabetic gastroparesis symptom severity diary (32), the gastroparesis cardinal symptom index (GCSI) (31), and the Nepean Dyspepsia Index (33). We believe that future trials should adopt such instruments and not rely on participant self-reporting of GI symptoms.

## Adverse consequences of motility effects of GLP-1RAs

In one study, the incidence of retained gastric contents in individuals using a GLP-1RA was 56%, compared with 19% for those not using GLP-1RAs (34), although notably there were more individuals with T2D and obesity in the group treated with GLP-1RAs. Supplemental Table 2 summarizes information relating to the risk for retained gastric contents and aspiration in association with upper GI endoscopy.

Three systematic reviews analyzing the risk of retained gastric contents causing premature termination of endoscopy have been published. Baig et al. (35) and Facciorusso et al. (36) reported an increased risk (odds ratios 4.5 and 5.6, respectively) for premature termination among individuals taking GLP-1RAs, while Singh et al. reported an even greater risk (odds ratio 13.9) (37). Importantly, none of these reviews identified an increased risk of aspiration pneumonia. However, it should be appreciated that this severe complication is rare and the risk is also less with endoscopy compared with surgical procedures that require general anesthesia (38). Population-based studies have reported mixed findings on the potential association between GLP-1RA use and aspiration pneumonia (39–44). Furthermore, a systematic review of randomized controlled trials and observational studies also failed to identify an increased risk of aspiration pneumonia in GLP-1RA users, although an increased risk of retained gastric contents was evident (45). In summary, GLP-1RA therapy is clearly associated with a substantially increased risk of retained gastric contents, but there is little evidence to suggest that this translates to an increased incidence of aspiration pneumonia.

Potential risks of retained gastric content and pulmonary aspiration have predictably raised the question as to whether GLP-1RA treatment should be discontinued or modified before procedures associated with such risk. Multi-society consensus guidelines have, in general, recommended an individualized approach toward the decision to withhold GLP-1RA therapy (46). For agents with a long elimination half-life (6, 7), typically used at more than 10 times higher than physiological concentrations, such periods probably would need to be much longer than a week. The maximum duration of any of the few studies in which a good technique has been used to quantify emptying (usually scintigraphy) is 16 weeks, and while the magnitude of the delay in emptying at that time point was not as marked as at 5 weeks, it was substantial in some cases (47). Other strategies have been suggested to reduce the risk of retained gastric contents. First, it has been observed that individuals on GLP-1RAs undergoing both endoscopy and colonoscopy had a much lower risk of retained gastric contents, suggesting that a prolonged period on a clear liquid diet may reduce this risk (48). Second, intravenous erythromycin (200 mg), which has been shown to abolish the slowing of gastric emptying by acute intravenous GLP-1 infusion (49), may represent a treatment for individuals identified at having retained gastric content via ultrasonography (50).

### Central and peripheral mechanisms underlying GI symptoms.

**Figure 1:**
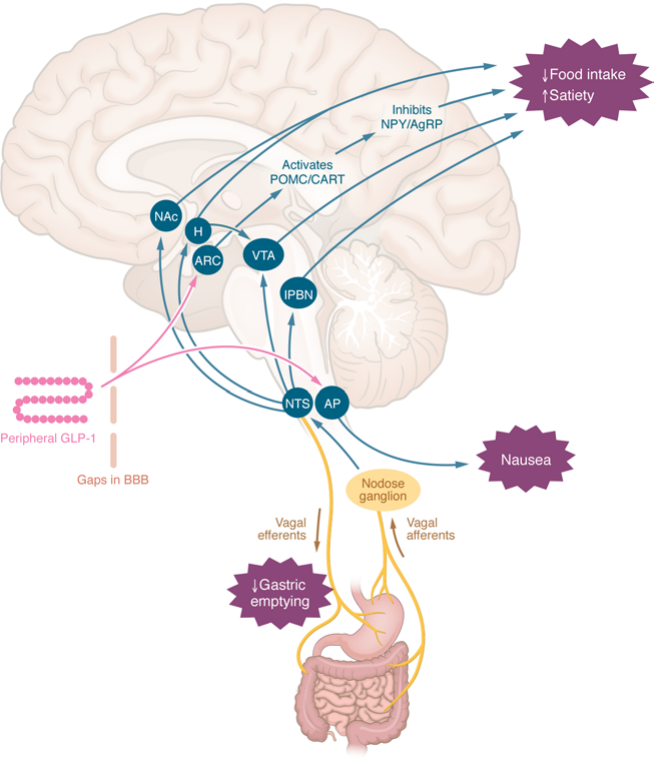
Central mechanisms by which peripheral GLP-1RAs may reduce appetite or induce nausea. Peripheral GLP-1 or GLP-1RAs may interact with appetite-regulating regions of the brain via gaps in the blood-brain barrier (27), via tanycyte uptake (32), or indirectly via the nodose ganglia (29). The effects to slow gastric emptying, induce nausea, and reduce energy intake are independent (34). AgRP, agouti-related peptide; AP, area postrema; ARC, arcuate nucleus; BBB, blood-brain barrier; CART, cocaine- and amphetamine-regulated transcript; H, hypothalamus; lPBN, lateral parabrachial nucleus; NAc, nucleus accumbens; NPY, neuropeptide Y; NTS, nucleus tractus solitarius; POMC, proopiomelanocortin; VTA, ventral tegmental area.

Nausea, vomiting, and diarrhea in association with GLP-1RA treatment are often referred to as GI adverse events, implying that they predominantly represent the expression of an altered GI functional state. Indeed, GLP-1R agonism slows gastric emptying (51, 52) and suppresses small intestinal motility (53). However, an alternative explanation, albeit not mutually exclusive, is direct interaction of GLP-1RAs with GLP-1R in brain regions such as the brainstem (area postrema, nucleus tractus solitarii), which are not protected by the blood-brain barrier, and are typically involved in medication-induced nausea (chemosensitive area) (54–56). Peripheral GLP-1/GLP-1RAs may also interact with the CNS indirectly by GLP-1R expressed on vagal afferent fibers, signaling via nodose ganglia (57) to the nucleus tractus solitarii within the brainstem. The nucleus tractus solitarii projects to multiple regions of the CNS involved in the regulation of appetite, including the hypothalamus, ventral tegmental area, lateral parabrachial nucleus, and nucleus accumbens (58, 59). Preclinical studies also indicate that tanycytes, specialized glial cells within the CNS, may facilitate the transportation of peripheral GLP-1RAs, such as semaglutide, into the CNS, bypassing the blood-brain barrier to access appetite-regulating hypothalamic regions such as the arcuate nucleus (60, 61). Importantly, there are central pathways that result in a reduction in energy intake without the induction of nausea (62). The mechanisms underlying the effect of GLP-1RAs on GI motility and symptoms like nausea and vomiting are summarized in Figure 1.

### Biliary/pancreatic complications.

In a systematic review of 55 double-blinded, placebo-controlled randomized clinical trials, GLP-1RA treatment was associated with an increased risk of cholelithiasis compared with placebo (RR 1.46, 95% CI 1.09–1.97) but not cholecystitis, cholangitis, or pancreatitis (63). This contrasts with case reports of acute pancreatitis with the first approved GLP-1RA, exenatide b.i.d. (64). The case reports of pancreatitis prompted searches of adverse event–reporting databases (65), which indicated a substantially higher risk for acute pancreatitis with GLP-1RA treatment. These searches have been reproduced with almost identical results (66), leading to the definition of acute pancreatitis as an “adverse event of special interest” in clinical trials of GLP-1RAs. The first trial reporting detailed results on adjudicated cases of suspected acute pancreatitis was the LEADER trial comparing liraglutide and placebo in patients with T2D (18 cases in 4668 participants randomized to liraglutide treatment vs. 23 in 4672 with placebo treatment) (67). Because of the suspected association with acute pancreatitis, amylase and lipase were measured throughout this trial (and in other clinical studies). Surprisingly, liraglutide treatment elevated serum amylase and lipase activity in the majority of participants (68), but such elevations did not predict clinical acute pancreatitis. Subsequent meta-analyses of cardiovascular outcomes trials with GLP-1RAs have clearly excluded a causal role for GLP-1RAs in acute pancreatitis (63, 69, 70), while therapy with inhibitors of dipeptidyl peptidase-4 may lead to a minor elevation of this risk (69). In retrospect, the suspicion that GLP-1RAs provoke acute pancreatitis can be traced to the diagnostic criteria used for acute pancreatitis (two out of the following three features: severe upper abdominal pain radiating into the back, elevations in amylase and/or lipase, and typical results of imaging procedures) and that GLP-1RA therapy induces abdominal symptoms in a large number of patients, as well as elevations in amylase and/or lipase. Careful adjudication of suspected cases was necessary to scrutinize details of the symptomatic presentation, the degree of elevation on pancreatic enzymes, and the diagnostic specificity of imaging data (68). Alarming reports published in 2011 (65) led to widely discussed safety concerns, which transiently limited the use of GLP-1RAs for the treatment of T2D. These events attest to the potential of adverse event–reporting databases for bias when a serious concern has achieved prominence. The quest for robust pharmacovigilance for suspected adverse events with relatively novel drugs remains an important issue, and benefits and risks must be weighed when available evidence is limited.

## Thyroid carcinomas

**Figure 2:**
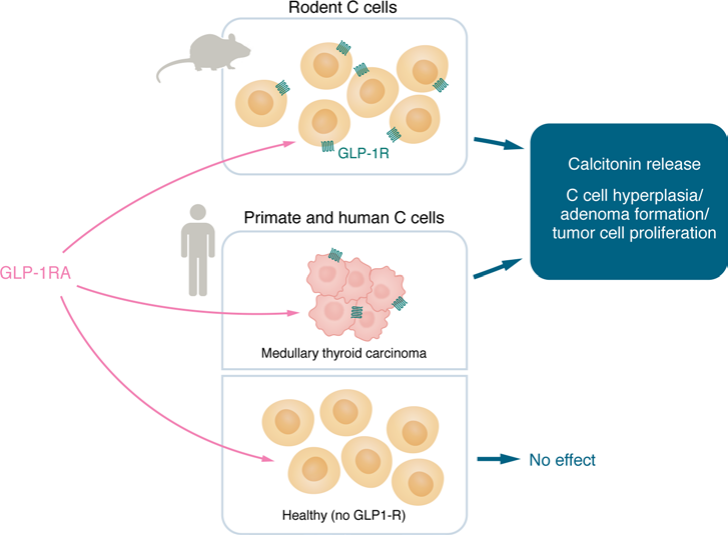
GLP-1Rs and thyroid C cells. GLP-1Rs are typically expressed in rodent C cells (83). Some GLP-1Rs are expressed in medullary thyroid cancer (85), but there are almost no GLP-1Rs are in healthy human/primary C cells (84). Accordingly, there may be an increased risk of medullary thyroid cancer with GLP-1RA use in susceptible individuals, but this may not be the case for healthy individuals.

Medullary thyroid carcinoma became a concern because of alerts from animal experiments showing increased calcitonin secretion and growth (hyperplasia, adenomas, and carcinomas) of C cells in rodents following treatment with the long-acting GLP-1RA liraglutide (71). The presence of GLP-1R in the rodent thyroid gland and on rodent C cells had been shown earlier (72, 73). In contrast, in primate and human thyroids, GLP-1R has either not been detected (74) or only found in small fractions of healthy C cells (75), and treatment with liraglutide does not stimulate calcitonin secretion (76). On the other hand, most human medullary thyroid carcinomas and hyperplastic C cells express GLP-1R (75, 77) (Figure 2). Accordingly, a personal or family history of medullary thyroid carcinoma or multiple endocrine neoplasia type 2 (MEN2) represents a contraindication to the use of GLP-1RAs and are exclusion criteria for participation in clinical trials with GLP-1RAs. Recent health insurance data from France are indicative of an elevated risk of medullary thyroid carcinoma in individuals treated with GLP-1RAs versus other glucose-lowering agents (35 cases, hazard ratio [HR] 1.78 [95% CI 1.04–3.05]) (78). A meta-analysis of cardiovascular outcomes trials indicates that spontaneous medullary thyroid carcinomas have been diagnosed in patients receiving GLP-1RA (6 with active vs. 2 with placebo treatment) (79). These findings reinforce the “at-risk” status as a contraindication to GLP-1RA treatment, but given their low incidence, screening measures to facilitate early diagnosis of medullary thyroid carcinomas in GLP-1RA–treated individuals have not been established.

Other histological types of thyroid carcinoma have not been examined as closely because less biological evidence exists for a convincing mechanism of action. GLP-1R has been detected in healthy thyroid cells other than C cells, as well as in some papillary thyroid cancers (75). A case-control study of health insurance data from France indicated an increased risk for thyroid carcinoma with GLP-1RA treatment, with an HR of 1.58 (95% CI 1.27–1.95) (78). In contrast, a Scandinavian cohort study using nationwide cancer registries found no association between GLP-1RA use and thyroid cancer (HR 0.93, 95 % CI 0.66–1.31) (80). Further information is needed before definitive recommendations can be made regarding GLP-1RA treatment in individuals with a family history of other thyroid cancers.

## Retinopathy and other vision-related complications

### Diabetic retinopathy.

**Figure 3:**
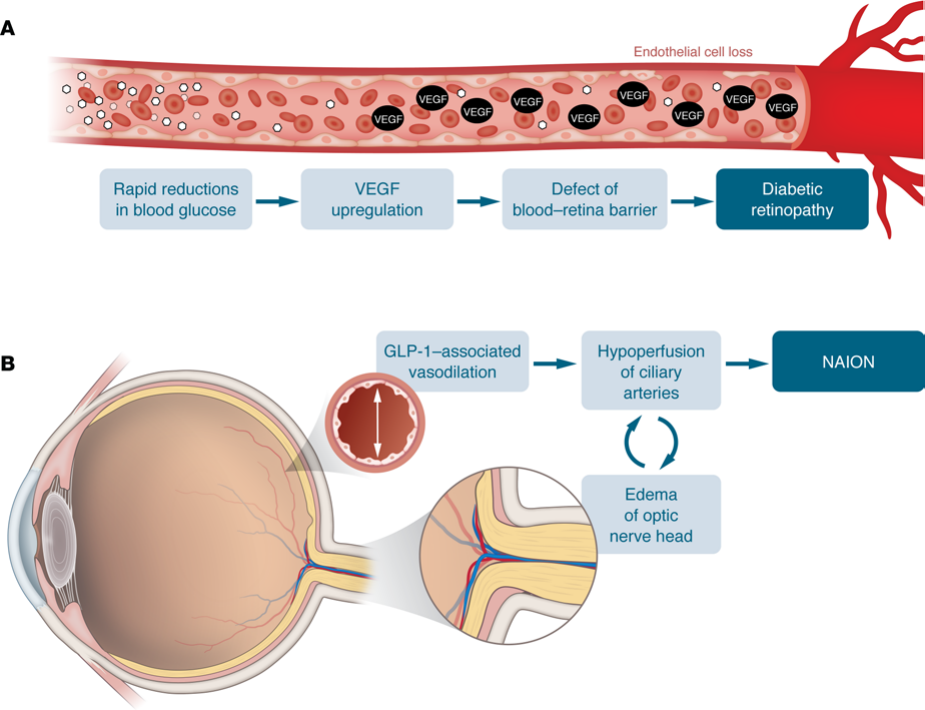
Hypothesized mechanisms for ocular complications associated with GLP-1RA therapy. In the eye, rapid reductions in glycemia due to GLP-1RA use may lead to the progression of diabetic retinopathy (**A**) or NAION (**B**). Figure based on Chou et al. (95) and Abdeen and le Roux (98).

Subcutaneous semaglutide treatment led to an increased number of retinopathy complications in the SUSTAIN-6 cardiovascular outcomes trial (81). “Retinopathy complications” were defined as a composite of vitreous hemorrhage, onset of diabetes-related blindness, and a need for treatment with intravitreal injection or retinal photocoagulation, all serious clinical events. In this study, baseline retinopathy status had, perhaps surprisingly, not been assessed, and there were not any systematic follow-up examinations during the trial. Semaglutide predictably resulted in greater reductions in both plasma glucose and HbA_1c_ concentrations than standard-of-care treatment. The observations in SUSTAIN-6 are predictable, given that intensified versus conventional insulin therapy is well known to have the capacity to precipitate initial worsening of retinopathy in patients with T2D with advanced preexisting retinopathy, as shown in the Diabetes Control and Complications Trial (82). However, in the longer term, retinopathy progression is slowed substantially with the improved glycemic control resulting from intensified insulin treatment (82) (Figure 3). Accordingly, a retrospective analysis of SUSTAIN-6 examined the role of preexisting advanced retinopathy and the induction of rapid reductions in plasma glucose and HbA_1c_ and found retinopathy complications mainly in those with preproliferative or proliferative retinopathy at baseline who experienced a large reduction in HbA_1c_ within 16 weeks after initiation of semaglutide (83). In SUSTAIN 1–5 and SUSTAIN 7, studies in which preexisting diabetic retinopathy and maculopathy were excluded, the rates of retinopathy were comparable across treatment groups (84). The same phenomenon was evident in those receiving other glucose-lowering medications (such as insulin or SGLT-2 inhibitors) as part of the standard of care (82, 85). A prospective trial focusing on the effects of semaglutide on retinopathy progression and complications (FOCUS) is ongoing (ClinicalTrials.gov NCT03811561; reporting in 2027) to further characterize risks and benefits associated with semaglutide treatment in T2D.

### Non-arteritic anterior ischemic optic neuropathy.

Non-arteritic anterior ischemic optic neuropathy (NAION) is a potential, but rare cause of blindness among adults (86). The pathophysiology of this condition is incompletely understood but thought to be related to hypoperfusion of the optic nerve head leading to edema and infarction of optic nerve fibers (87, 88). Hathaway et al. reported in a cohort study (*n* = 16,827) that there was a higher risk of NAION in individuals prescribed semaglutide (HR 4.28) (89). Cai et al. subsequently confirmed this association in a large multicenter database study of 37.1 million adults, but the incidence was low at 14.5 per 100,000 person-years among semaglutide users, and there was only a modest increase in risk attributable to semaglutide (90). Grausland et al. in a 5-year longitudinal cohort study found a stronger association, reporting that semaglutide exposure more than doubled the risk of NAION (91). Fung et al. specifically examined those older than 65 years with T2D and also found an association between semaglutide and NAION (HR 1.39, 95% CI 1.13–1.72) (92). None of these studies have been able to establish causality, and complicated T2D and hypertension are known risk factors for NAION (93). The association may, therefore, reflect the higher presence of risk factors in semaglutide users rather than an effect of semaglutide. Furthermore, some studies have not found any increase in the risk of NAION in individuals using semaglutide or other GLP-1RAs (94, 95). Dedicated, prospective studies are required to confirm or refute this association, to clarify causality, and to determine whether there is a class effect with all GLP-1RAs. The mechanism(s) by which GLP-1RAs would cause NAION is also unclear, particularly as GLP-1RAs are associated with neuroprotective properties and reductions in ischemic risk (96), but it has been hypothesized to be related, like the exacerbation of retinopathy, to rapid improvements in glycemic control (Figure 3).

## Potential psychiatric complications GLP-1R agonism

Chronic diseases, including diabetes and obesity, are risk factors for depression and suicidal ideation (97). Furthermore, treatment of T2D and obesity with metabolic surgery, which results in increased GLP-1 secretion (98), has been associated with an increased risk of suicide and self-harm (99), hinting to a potential causal relationship. Along those lines, Kornelius et al. reported a three-fold higher risk of major depression, and a doubled risk for anxiety and suicidal behavior in a large (*n* = 11,683,623) retrospective study of GLP-1RA users utilizing the TrinetX database (100). However, another systematic review by Chen et al. (101) found the opposite; GLP-1RA use was associated with a reduction in depression, suggesting that GLP-1RA could potentially be considered an antidepressant therapy. In two recent systematic reviews and meta-analyses, no link between GLP-1RA use (for obesity and/or T2D) and increased suicidal ideation was identified, but there was substantial heterogeneity between studies, particularly regarding the definition of suicidal ideation (102, 103). Furthermore, many of these were pharmacovigilance studies, and the outcome of suicidal ideation was inconsistently documented (104). The best designed study to date is a systematic review of 80 randomized clinical trials involving 107,860 participants, which found no association with GLP-1RA treatment and serious psychiatric adverse effects: major depression, suicide, or psychosis (105). Furthermore, GLP-1RA therapy was associated with an improvement in mental health–related quality of life (105). Accordingly, recent systematic reviews have provided substantial reassurance regarding the psychiatric safety profile of GLP-1RAs.

## GLP-1 and adverse effects in specific populations

### Younger individuals.

In a meta-analysis of 5 studies evaluating GLP-1RAs in younger individuals with T2D, there was an increased incidence of adverse effects, but withdrawal rates remained low (106). Children and adolescents with obesity are at an increased risk of disordered eating behaviors or eating disorders (107). A small, open-label retrospective cohort study reported reductions in Binge Eating Scale scores for individuals managed with semaglutide (108), but larger trials are needed.

### Pregnancy.

In animal studies, GLP-1RA exposure was associated with reduced fetal growth, delayed skeletal ossification, and reductions in maternal weight gain and food consumption (109). Therefore, it is not recommended that individuals who are planning pregnancy or currently pregnant use GLP-1RAs. In one relatively small study (*n* = 168), GLP-1RA use was not associated with an increased risk of pregnancy loss or birth defects in humans (110). Larger studies are needed to confirm this, although these would be challenging or infeasible to conduct.

### Advanced hepatic disease/hepatic functional impairment.

In a population-based study of 467 matched pairs of GLP-1RA users and non-users with T2D and liver cirrhosis, GLP-1RA use was associated with lower risks of death, cardiovascular events, decompensated cirrhosis, hepatic encephalopathy, and liver failure (101). In a cohort study of individuals with T2D and metabolic-associated fatty liver cirrhosis (459 GLP-1RA users), GLP-1RA therapy was associated with reduced risks of hepatic decompensation, portal hypertension, hepatocellular carcinoma, and liver transplantation (111). While these observations are supportive of GLP-1RA use in liver cirrhosis, prospective studies are needed to confirm this. The odds ratio for adverse GI events (e.g., nausea, vomiting, diarrhea, constipation) in individuals with metabolic dysfunction–associated steatotic liver disease using GLP-1RA versus placebo/other diabetes therapy was 4.83 (95% CI 3.36–6.95) (112), which is comparable to the results summarized in Table 2. There was no increased rate of adverse effects leading to discontinuation of treatment in GLP-1RA users compared with non–GLP-1RA users (112).

### Advanced renal disease/severe renal functional impairment.

GI adverse effects are more common in individuals with end-stage renal disease using GLP-1RAs (113), as is hypoglycemia in those using concomitant insulin (114). These adverse effects must be weighed against the potential benefits of GLP-1RAs. In a large cohort study of individuals with T2D and stage 5 chronic kidney disease (*n* = 27,279), GLP-1RA use was associated with lower all-cause mortality (HR 0.79; 95% CI 0.63–0.98) and reduced risks of sepsis and infection-related mortality compared with those using dipeptidyl peptidase-4 inhibitors (115). In small studies, GLP-1RAs resulted in modest weight loss in end-stage renal disease and renal transplant recipients (113). Large-scale prospective studies for GLP-1RA use in end-stage renal disease are awaited.

### Older individuals.

A claims-based database study indicated that the rate of discontinuation of GLP-1RAs in older individuals was high. After 24 months of follow-up, 68.2% of individuals less than 65 years old had discontinued GLP-1RAs, with the proportion rising to 75.3% for those 65–74 years old and 82.6% for those 75 or older (116). Possible reasons for the high rates of discontinuation include cost, variable efficacy, patient preferences, and/or GI adverse effects. However, in a study of weekly dulaglutide, there was a comparable incidence of GI adverse effects in those above and below 65 years of age (117). Another potential risk of relevance to older individuals is bone loss, which was observed with semaglutide (118), although combined treatment with an exercise program may potentially mitigate this risk (119). These “age-related” risks also must be balanced against the cardiovascular risk reduction associated with GLP-1RA use. In a systematic review, three-component Major Adverse Cardiovascular Events (3-p MACE) was reduced, with an HR of 0.86 (95% CI 0.80–0.92) for those over 65 years old, and there was an even more pronounced effect in those over age 75 (HR 0.75, 95% CI 0.61–0.92) (120). Older individuals are also at risk of sarcopenia, as discussed below.

### Reduced muscle mass.

With weight loss, there is also loss of fat-free mass, which raises concern for the development of sarcopenia. The proportion of total weight loss that was fat-free mass was 39% with semaglutide and 25% with tirzepatide (121). However, there are limitations inherent with this method as a surrogate to evaluating loss of muscle mass (few studies specifically measure muscle mass). For example, with weight reduction, liver mass may decrease, and this would be observed as a reduction in fat-free mass. It is also not known if this loss of fat-free mass is associated with reduced physical functioning. In contrast, a recent study indicated that muscle quality may be improved with tirzepatide therapy, as there is less intramuscular fat infiltration (122). Furthermore, the reduced mechanical load resulting from GLP-1RA–associated weight loss may improve aspects of physical function, as shown in the STEP 1–4 trials involving semaglutide (123). However, to date there are no studies assessing functional consequences, such as timed sit-to-stand tests. Due to reductions in appetite associated with GLP-1RA therapy, there is also the potential for consumption of dietary protein to be inadequate. Use of protein supplements has not yet been adequately evaluated in individuals on GLP-1RAs, but there is evidence that an exercise program may increase the proportion of fat mass loss, as opposed to loss of fat-free mass (124).

## Summary and outlook

A summary of the relative risk of adverse events associated with GLP-1RAs is presented in Table 1. Given the role of GLP-1RAs and other incretin-based medications widely used for the treatment of T2D and obesity, surprisingly little research has been directed at a comprehensive assessment of even the most frequent GI adverse events (125). Based on this suboptimal documentation, potential differences in the risks for such adverse events between compounds/preparations are often not appreciated. Given the vast number of incretin-based medications under development, mainly aiming at higher efficacy in body weight reduction, one may overlook that adherence to, and persistence of, therapy with GLP-1RAs in real-world studies is relatively low (18, 126). Improved methods to assess the adverse events associated with GLP-1RA medications should be introduced. Pharmacovigilance databases have, not surprisingly, been prone to reporting bias, once case reports have alerted the community to potentially important adverse events. For clinical trials, particularly during early development phases where safety and tolerability are the main focus, better tools should facilitate the identification of optimized dose-escalation regimens. Given the large and increasing number of currently developed novel agents within the class of GLP-1RAs (127, 128), it can be anticipated that there will be increasing competition to achieve better tolerability and safety.

## Funding support

- Royal Adelaide Hospital and Mary Overton Early Career Fellowship grant ID 18815 (to RJJ).

## Supplementary Material

Supplemental data

## Figures and Tables

**Table 3:**
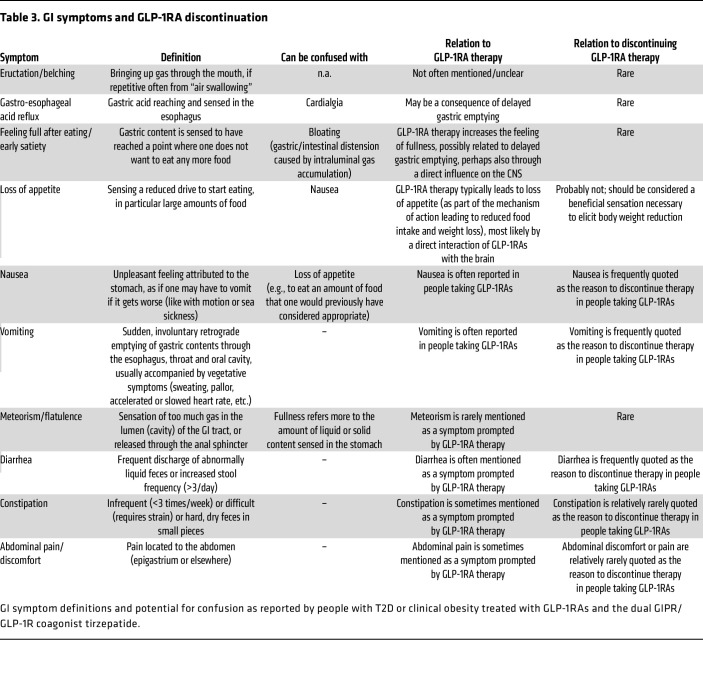
GI symptoms and GLP-1RA discontinuation

**Table 1:**
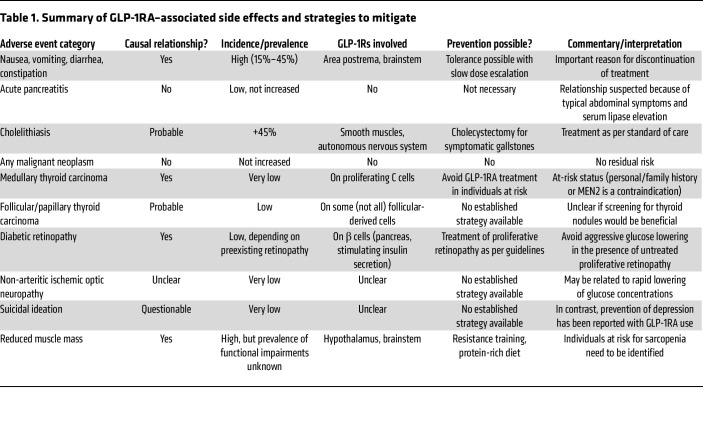
Summary of GLP-1RA–associated side effects and strategies to mitigate

**Table 2:**
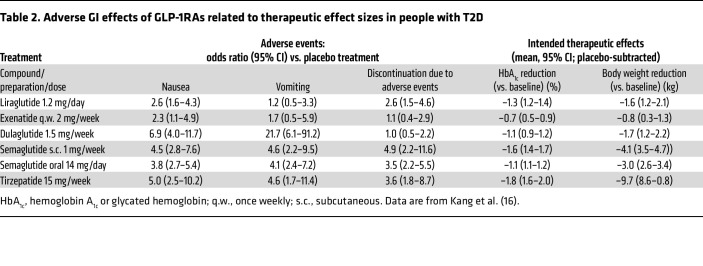
Adverse GI effects of GLP-1RAs related to therapeutic effect sizes in people with T2D

**Table 4:**
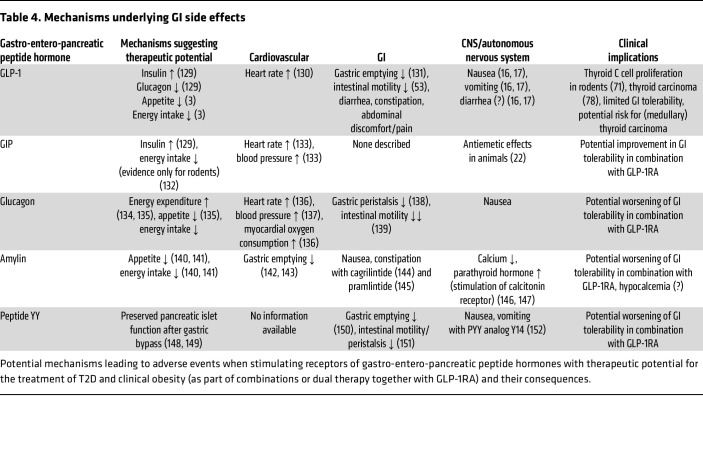
Mechanisms underlying GI side effects

## Version 1 02/16/2026

Electronic publication

## References

[1] Nauck MA (1993). Preserved incretin activity of glucagon-like peptide 1 [7-36 amide] but not of synthetic human gastric inhibitory polypeptide in patients with type-2 diabetes mellitus. J Clin Invest.

[2] Nauck MA (1993). Normalization of fasting hyperglycaemia by exogenous glucagon-like peptide 1 (7-36 amide) in type 2 (non-insulin-dependent) diabetic patients. Diabetologia.

[3] Flint A (1998). Glucagon-like peptide 1 promotes satiety and suppresses energy intake in humans. J Clin Invest.

[4] Mentis N (2011). GIP does not potentiate the antidiabetic effects of GLP-1 in hyperglycemic patients with type 2 diabetes. Diabetes.

[5] Drucker DJ, Nauck MA (2006). The incretin system: glucagon-like peptide-1 receptor agonists and dipeptidyl peptidase-4 inhibitors in type 2 diabetes. Lancet.

[6] Nauck MA, Meier JJ (2019). Management of endocrine disease: are all GLP-1 agonists equal in the treatment of type 2 diabetes?. Eur J Endocrinol.

[7] Nauck MA (2021). GLP-1 receptor agonists in the treatment of type 2 diabetes - state-of-the-art. Mol Metab.

[8] Thorens B (1992). Expression cloning of the pancreatic beta cell receptor for the gluco-incretin hormone glucagon-like peptide 1. Proc Natl Acad Sci U S A.

[9] Wei Y, Mojsov S (1995). Tissue-specific expression of the human receptor for glucagon-like peptide-I: brain, heart and pancreatic forms have the same deduced amino acid sequences. FEBS Lett.

[10] Alvarez E (1996). Expression of the glucagon-like peptide-1 receptor gene in rat brain. J Neurochem.

[11] Pyke C, Knudsen LB (2013). The glucagon-like peptide-1 receptor--or not?. Endocrinology.

[12] McLean BA (2021). Revisiting the complexity of GLP-1 action from sites of synthesis to receptor activation. Endocr Rev.

[13] Hebsgaard JB (2018). Glucagon-like peptide-1 receptor expression in the human eye. Diabetes Obes Metab.

[14] Drucker DJ (2024). Efficacy and safety of GLP-1 medicines for type 2 diabetes and obesity. Diabetes Care.

[15] Ismaiel A (2025). Gastrointestinal adverse events associated with GLP-1 RA in non-diabetic patients with overweight or obesity: a systematic review and network meta-analysis. Int J Obes (Lond).

[16] Kang YM (2025). Comparative efficacy and tolerability of currently approved incretin mimetics: A systematic analysis of placebo-controlled clinical trials. Diabetes Obes Metab.

[17] Bettge K (2017). Occurrence of nausea, vomiting and diarrhoea reported as adverse events in clinical trials studying glucagon-like peptide-1 receptor agonists: A systematic analysis of published clinical trials. Diabetes Obes Metab.

[18] Weeda ER (2021). Medication adherence to injectable glucagon-like peptide-1 (GLP-1) receptor agonists dosed once weekly vs once daily in patients with type 2 diabetes: a meta-analysis. Int J Clin Pract.

[19] Wilke T (2016). Non-persistence and non-adherence of patients with type 2 diabetes mellitus in therapy with GLP-1 receptor agonists: a retrospective analysis. Diabetes Ther.

[20] Eldor R (2025). Gradual titration of semaglutide results in better treatment adherence and fewer adverse events: a randomized controlled open-label pilot study examining a 16-week flexible titration regimen versus label-recommended 8-week semaglutide titration regimen. Diabetes Care.

[21] Aroda VR (2025). High-dose semaglutide (up to 16 mg) in people with type 2 diabetes and overweight or obesity: a randomized, placebo-controlled, phase 2 trial. Diabetes Care.

[22] Borner T (2021). GIP receptor agonism attenuates GLP-1 receptor agonist-induced nausea and emesis in preclinical models. Diabetes.

[23] Knop FK (2024). A long-acting glucose-dependent insulinotropic polypeptide receptor agonist improves the gastrointestinal tolerability of glucagon-like peptide-1 receptor agonist therapy. Diabetes Obes Metab.

[24] Frias JP (2021). Tirzepatide versus semaglutide once weekly in patients with type 2 diabetes. N Engl J Med.

[25] Nicholls S Tirzepatide, dulaglutide, and cardiovascular outcomes in high cardiovascular risk patients with type 2 diabetes. N Engl J Med.

[26] Du YT (2018). Gastrointestinal symptoms in diabetes: prevalence, assessment, pathogenesis, and management. Diabetes Care.

[27] de Kort S (2012). Gastrointestinal symptoms in diabetes mellitus, and their relation to anxiety and depression. Diabetes Res Clin Pract.

[28] Ang D (2011). Review article: Endpoints used in functional dyspepsia drug therapy trials. Aliment Pharmacol Ther.

[29] Talley NJ (1989). A patient questionnaire to identify bowel disease. Ann Intern Med.

[30] Quan C (2003). Development and validation of the diabetes bowel symptom questionnaire. Aliment Pharmacol Ther.

[31] Rentz AM (2004). Development and psychometric evaluation of the patient assessment of upper gastrointestinal symptom severity index (PAGI-SYM) in patients with upper gastrointestinal disorders. Qual Life Res.

[32] Fehnel S (2019). Development and psychometric evaluation of the Diabetic Gastroparesis Symptom Severity Diary. Clin Exp Gastroenterol.

[33] Jones MP (2019). The Nepean Dyspepsia Index is a valid instrument for measuring quality-of-life in functional dyspepsia. Eur J Gastroenterol Hepatol.

[34] Sen S (2024). Glucagon-like peptide-1 receptor agonist use and residual gastric content before anesthesia. JAMA Surg.

[35] Baig MU (2025). Glucagon-like peptide-1 receptor agonist use and the risk of residual gastric contents and aspiration in patients undergoing GI endoscopy: a systematic review and a meta-analysis. Gastrointest Endosc.

[36] Facciorusso A (2025). Effects of glucagon-like peptide-1 receptor agonists on upper gastrointestinal endoscopy: a meta-analysis. Clin Gastroenterol Hepatol.

[37] Singh S (2025). Effects of glucagon-like peptide-1 receptor agonists on endoscopy outcomes: systematic review and meta-analysis. Gastrointest Endosc.

[38] Green SM (2017). Pulmonary aspiration during procedural sedation: a comprehensive systematic review. Br J Anaesth.

[39] Yeo YH (2024). Increased risk of aspiration pneumonia associated with endoscopic procedures among patients with glucagon-like peptide 1 receptor agonist use. Gastroenterology.

[40] Al Sakka Amini R (2024). Risk of aspiration pneumonitis after elective esophagogastroduodenoscopy in patients on glucagon-like peptide-1 receptor agonists. Cureus.

[41] Chen YH (2025). Postoperative aspiration pneumonia among adults using GLP-1 receptor agonists. JAMA Netw Open.

[42] Barlowe TS (2024). Glucagon-like peptide-1 receptor agonists do not increase aspiration during upper endoscopy in patients with diabetes. Clin Gastroenterol Hepatol.

[43] Dixit AA (2024). Preoperative GLP-1 receptor agonist use and risk of postoperative respiratory complications. JAMA.

[44] Velji-Ibrahim J (2025). GLP-1 receptor agonist use does not increase risk of respiratory complications post-endoscopy. Endosc Int Open.

[45] Elkin J (2025). Association between glucagon-like peptide-1 receptor agonist use and peri-operative pulmonary aspiration: a systematic review and meta-analysis. Anaesthesia.

[46] Kindel TL (2025). Multi-society clinical practice guidance for the safe use of glucagon-like peptide-1 receptor agonists in the perioperative period. Surg Endosc.

[47] Jalleh RJ (2024). Clinical consequences of delayed gastric emptying with GLP-1 receptor agonists and tirzepatide. J Clin Endocrinol Metab.

[48] Silveira SQ (2023). Relationship between perioperative semaglutide use and residual gastric content: A retrospective analysis of patients undergoing elective upper endoscopy. J Clin Anesth.

[49] Meier JJ (2005). Erythromycin antagonizes the deceleration of gastric emptying by glucagon-like peptide 1 and unmasks its insulinotropic effect in healthy subjects. Diabetes.

[50] Jalleh RJ (2024). Gastrointestinal effects of GLP-1 receptor agonists: mechanisms, management, and future directions. Lancet Gastroenterol Hepatol.

[51] Meier JJ (2015). Contrasting effects of lixisenatide and liraglutide on postprandial glycemic control, gastric emptying, and safety parameters in patients with type 2 diabetes on optimized insulin glargine with or without metformin: a randomized, open-label trial. Diabetes Care.

[52] Meier JJ (2012). GLP-1 receptor agonists for individualized treatment of type 2 diabetes mellitus. Nat Rev Endocrinol.

[53] Hellström PM (2008). GLP-1 suppresses gastrointestinal motility and inhibits the migrating motor complex in healthy subjects and patients with irritable bowel syndrome. Neurogastroenterol Motil.

[54] Baumgartner I (2010). Hepatic-portal vein infusions of glucagon-like peptide-1 reduce meal size and increase c-Fos expression in the nucleus tractus solitarii, area postrema and central nucleus of the amygdala in rats. J Neuroendocrinol.

[55] Kawatani M (2018). Glucagon-like peptide-1 (GLP-1) action in the mouse area postrema neurons. Peptides.

[56] Lu Z (2017). Centrally located GLP-1 receptors modulate gastric slow waves and cardiovascular function in ferrets consistent with the induction of nausea. Neuropeptides.

[57] Krieger JP (2016). Knockdown of GLP-1 receptors in vagal afferents affects normal food intake and glycemia. Diabetes.

[58] Alhadeff AL (2012). GLP-1 neurons in the nucleus of the solitary tract project directly to the ventral tegmental area and nucleus accumbens to control for food intake. Endocrinology.

[59] Richard JE (2014). GLP-1 receptor stimulation of the lateral parabrachial nucleus reduces food intake: neuroanatomical, electrophysiological, and behavioral evidence. Endocrinology.

[60] Gabery S (2020). Semaglutide lowers body weight in rodents via distributed neural pathways. JCI Insight.

[61] Secher A (2014). The arcuate nucleus mediates GLP-1 receptor agonist liraglutide-dependent weight loss. J Clin Invest.

[62] Kanoski SE (2016). GLP-1 and weight loss: unraveling the diverse neural circuitry. Am J Physiol Regul Integr Comp Physiol.

[63] Chiang CH (2025). Glucagon-like peptide-1 receptor agonists and gastrointestinal adverse events: a systematic review and meta-analysis. Gastroenterology.

[64] Ayoub WA (2011). Exenatide-induced acute pancreatitis. Endocr Pract.

[65] Elashoff M (2011). Pancreatitis, pancreatic, and thyroid cancer with glucagon-like peptide-1-based therapies. Gastroenterology.

[66] Nauck MA, Friedrich N (2013). Do GLP-1-based therapies increase cancer risk?. Diabetes Care.

[67] Marso SP (2016). Liraglutide and cardiovascular outcomes in type 2 diabetes. N Engl J Med.

[68] Steinberg WM (2017). Amylase, lipase, and acute pancreatitis in people with type 2 diabetes treated with liraglutide: results from the LEADER randomized trial. Diabetes Care.

[69] Abd El Aziz M (2020). Incretin-based glucose-lowering medications and the risk of acute pancreatitis and malignancies: a meta-analysis based on cardiovascular outcomes trials. Diabetes Obes Metab.

[70] Nauck MA (2017). Incretin-based glucose-lowering medications and the risk of acute pancreatitis and/or pancreatic cancer: Reassuring data from cardio-vascular outcome trials. Diabetes Obes Metab.

[71] Bjerre Knudsen L (2010). Glucagon-like peptide-1 receptor agonists activate rodent thyroid C-cells causing calcitonin release and C-cell proliferation. Endocrinology.

[72] Crespel A (1996). Effects of glucagon and glucagon-like peptide-1-(7-36) amide on C cells from rat thyroid and medullary thyroid carcinoma CA-77 cell line. Endocrinology.

[73] Vertongen P (1994). Pituitary adenylate cyclase-activating polypeptide receptors of types I and II and glucagon-like peptide-I receptors are expressed in the rat medullary carcinoma of the thyroid cell line 6/23. Endocrinology.

[74] Pyke C (2014). GLP-1 receptor localization in monkey and human tissue: novel distribution revealed with extensively validated monoclonal antibody. Endocrinology.

[75] Gier B (2012). Glucagon like peptide-1 receptor expression in the human thyroid gland. J Clin Endocrinol Metab.

[76] Hegedüs L (2011). GLP-1 and calcitonin concentration in humans: lack of evidence of calcitonin release from sequential screening in over 5000 subjects with type 2 diabetes or nondiabetic obese subjects treated with the human GLP-1 analog, liraglutide. J Clin Endocrinol Metab.

[77] Pach D (2013). Glucagon-like peptide-1 receptor imaging with [Lys (40) (Ahx-HYNIC- (99 m) Tc/EDDA)NH 2]-exendin-4 for the diagnosis of recurrence or dissemination of medullary thyroid cancer: a preliminary report. Int J Endocrinol.

[78] Bezin J (2023). GLP-1 receptor agonists and the risk of thyroid cancer. Diabetes Care.

[79] Lee MMY (2025). Cardiovascular and kidney outcomes and mortality with long-acting injectable and oral glucagon-like peptide 1 receptor agonists in individuals with type 2 diabetes: a systematic review and meta-analysis of randomized trials. Diabetes Care.

[80] Pasternak B (2024). Glucagon-like peptide 1 receptor agonist use and risk of thyroid cancer: Scandinavian cohort study. BMJ.

[81] Marso SP (2016). Semaglutide and cardiovascular outcomes in patients with type 2 diabetes. N Engl J Med.

[82] Diabetes Control Coplications Trial Research Group (1993). The effect of intensive treatment of diabetes on the development and progression of long-term complications in insulin-dependent diabetes mellitus. N Engl J Med.

[83] Vilsbøll T (2018). Semaglutide, reduction in glycated haemoglobin and the risk of diabetic retinopathy. Diabetes Obes Metab.

[84] Aroda VR (2019). Comparative efficacy, safety, and cardiovascular outcomes with once-weekly subcutaneous semaglutide in the treatment of type 2 diabetes: Insights from the SUSTAIN 1-7 trials. Diabetes Metab.

[85] Joo JH (2024). The effect of glucagon-like peptide-1 receptor agonists on diabetic retinopathy at a tertiary care center. Ophthalmol Sci.

[86] Johnson LN, Arnold AC (1994). Incidence of nonarteritic and arteritic anterior ischemic optic neuropathy. Population-based study in the state of Missouri and Los Angeles County, California. J Neuroophthalmol.

[87] Sharma RA (2019). New concepts on acute ocular ischemia. Curr Opin Neurol.

[88] (2019). Unraveling the enigma of nonarteritic anterior ischemic optic neuropathy. J Neuroophthalmol.

[89] Hathaway JT (2024). Risk of nonarteritic anterior ischemic optic neuropathy in patients prescribed semaglutide. JAMA Ophthalmol.

[90] Cai CX (2025). Semaglutide and nonarteritic anterior ischemic optic neuropathy. JAMA Ophthalmol.

[91] Grauslund J (2024). Once-weekly semaglutide doubles the five-year risk of nonarteritic anterior ischemic optic neuropathy in a Danish cohort of 424,152 persons with type 2 diabetes. Int J Retina Vitreous.

[92] Fung KW (2025). GLP-1 RAs and risk of nonarteritic anterior ischemic optic neuropathy in older patients with diabetes. JAMA Ophthalmol.

[93] Cestari DM (2016). Demographic, systemic, and ocular factors associated with nonarteritic anterior ischemic optic neuropathy. Ophthalmology.

[94] Abbass NJ (2025). The effect of semaglutide and GLP-1 RAs on risk of nonarteritic anterior ischemic optic neuropathy. Am J Ophthalmol.

[95] Chou CC (2025). Association between semaglutide and nonarteritic anterior ischemic optic neuropathy: a multinational population-based study. Ophthalmology.

[96] Yang X (2022). Neuroprotective mechanisms of glucagon-like peptide-1-based therapies in ischemic stroke: an update based on preclinical research. Front Neurol.

[97] Luppino FS (2010). Overweight, obesity, and depression: a systematic review and meta-analysis of longitudinal studies. Arch Gen Psychiatry.

[98] Abdeen G, le Roux CW (2016). Mechanism underlying the weight loss and complications of roux-en-y gastric bypass. Review. Obes Surg.

[99] Castaneda D (2019). Risk of suicide and self-harm is increased after bariatric surgery-a systematic review and meta-analysis. Obes Surg.

[100] Kornelius E (2024). The risk of depression, anxiety, and suicidal behavior in patients with obesity on glucagon like peptide-1 receptor agonist therapy. Sci Rep.

[101] Yen FS (2024). Glucagon-like peptide-1 receptor agonist use in patients with liver cirrhosis and type 2 diabetes. Clin Gastroenterol Hepatol.

[102] Bushi G (2025). Association of GLP-1 receptor agonists with risk of suicidal ideation and behaviour: a systematic review and meta-analysis. Diabetes Metab Res Rev.

[103] Ebrahimi P (2025). Suicide and self-harm events with GLP-1 receptor agonists in adults with diabetes or obesity: a systematic review and meta-analysis. JAMA Psychiatry.

[104] Anderson HD (2015). Monitoring suicidal patients in primary care using electronic health records. J Am Board Fam Med.

[105] Pierret ACS (2025). Glucagon-like peptide 1 receptor agonists and mental health: a systematic review and meta-analysis. JAMA Psychiatry.

[106] Yugar LBT (2024). The efficacy and safety of GLP-1 receptor agonists in youth with type 2 diabetes: a meta-analysis. Diabetol Metab Syndr.

[107] Stabouli S (2021). Obesity and eating disorders in children and adolescents: the bidirectional link. Nutrients.

[108] Richards J (2023). Successful treatment of binge eating disorder with the GLP-1 agonist semaglutide: A retrospective cohort study. Obes Pillars.

[109] Muller DRP (2023). Effects of GLP-1 agonists and SGLT2 inhibitors during pregnancy and lactation on offspring outcomes: a systematic review of the evidence. Front Endocrinol (Lausanne).

[110] Dao K (2024). Use of GLP1 receptor agonists in early pregnancy and reproductive safety: a multicentre, observational, prospective cohort study based on the databases of six Teratology Information Services. BMJ Open.

[111] Elsaid MI (2024). Impacts of glucagon-like peptide-1 receptor agonists on the risk of adverse liver outcomes in patients with metabolic dysfunction-associated steatotic liver disease cirrhosis and type 2 diabetes. Aliment Pharmacol Ther.

[112] Huang X (2025). Gastrointestinal adverse events associated with GLP-1 receptor agonists in metabolic dysfunction-associated steatotic liver disease (MASLD): a systematic review and meta-analysis. Front Med (Lausanne).

[113] Clemens KK (2023). Glucagon-like peptide 1 receptor agonists in end-staged kidney disease and kidney transplantation: a narrative review. Nutr Metab Cardiovasc Dis.

[114] Thomas AM (2023). Glucagon-like peptide-1 receptor agonists use for type 2 diabetes mellitus in end-stage renal disease. J Am Pharm Assoc (2003).

[115] Chen JJ (2022). Association of glucagon-like peptide-1 receptor agonist vs dipeptidyl peptidase-4 inhibitor use with mortality among patients with type 2 diabetes and advanced chronic kidney disease. JAMA Netw Open.

[116] Weiss T (2020). Real-world adherence and discontinuation of glucagon-like peptide-1 receptor agonists therapy in type 2 diabetes mellitus patients in the United States. Patient Prefer Adherence.

[117] Boustani MA (2016). Similar efficacy and safety of once-weekly dulaglutide in patients with type 2 diabetes aged ≥65 and <65 years. Diabetes Obes Metab.

[118] Hansen MS (2024). Once-weekly semaglutide versus placebo in adults with increased fracture risk: a randomised, double-blinded, two-centre, phase 2 trial. EClinicalMedicine.

[119] Jensen SBK (2024). Bone health after exercise alone, GLP-1 receptor agonist treatment, or combination treatment: a secondary analysis of a randomized clinical trial. JAMA Netw Open.

[120] Karagiannis T (2021). GLP-1 receptor agonists and SGLT2 inhibitors for older people with type 2 diabetes: a systematic review and meta-analysis. Diabetes Res Clin Pract.

[121] Conte C (2024). Is weight loss-induced muscle mass loss clinically relevant?. JAMA.

[122] Sattar N (2025). Tirzepatide and muscle composition changes in people with type 2 diabetes (SURPASS-3 MRI): a post-hoc analysis of a randomised, open-label, parallel-group, phase 3 trial. Lancet Diabetes Endocrinol.

[123] Rubino D (2024). Effect of semaglutide 2.4 mg on physical functioning and weight- and health-related quality of life in adults with overweight or obesity: patient-reported outcomes from the STEP 1-4 trials. Diabetes Obes Metab.

[124] Lundgren JR (2021). Healthy weight loss maintenance with exercise, liraglutide, or both combined. N Engl J Med.

[125] Jalleh RJ (2023). Accurate measurements of gastric emptying and gastrointestinal symptoms in the evaluation of glucagon-like peptide-1 receptor agonists. Ann Intern Med.

[126] Mody R (2019). Adherence, persistence, glycaemic control and costs among patients with type 2 diabetes initiating dulaglutide compared with liraglutide or exenatide once weekly at 12-month follow-up in a real-world setting in the United States. Diabetes Obes Metab.

[127] Kusminski CM (2024). Transforming obesity: the advancement of multi-receptor drugs. Cell.

[128] Nogueiras R (2023). Gut hormone co-agonists for the treatment of obesity: from bench to bedside. Nat Metab.

[129] Nauck MA, Müller TD (2023). Incretin hormones and type 2 diabetes. Diabetologia.

[130] Lubberding AF (2024). Glucagon-like peptide-1 increases heart rate by a direct action on the sinus node. Cardiovasc Res.

[131] Wettergren A (1993). Truncated GLP-1 (proglucagon 78-107-amide) inhibits gastric and pancreatic functions in man. Dig Dis Sci.

[132] Zhang Q (2021). The glucose-dependent insulinotropic polypeptide (GIP) regulates body weight and food intake via CNS-GIPR signaling. Cell Metab.

[133] Heimbürger SM (2020). Glucose-dependent insulinotropic polypeptide (GIP) and cardiovascular disease. Peptides.

[134] Kleinert M (2019). Glucagon regulation of energy expenditure. Int J Mol Sci.

[135] Al-Massadi O (2019). Glucagon control on food intake and energy balance. Int J Mol Sci.

[136] Manchester JH (1970). Effects of glucagon on myocardial oxygen consumption and coronary blood flow in man and in dog. Circulation.

[137] (1969). Glucagon and the cardiovascular system. Ann Intern Med.

[138] Mochiki E (1998). Mechanism of inhibitory effect of glucagon on gastrointestinal motility and cause of side effects of glucagon. J Gastroenterol.

[139] Lahoti S (1997). A prospective, double-blind trial of L-hyoscyamine versus glucagon for the inhibition of small intestinal motility during ERCP. Gastrointest Endosc.

[140] Coester B (2020). RAMP1 and RAMP3 differentially control amylin’s effects on food intake, glucose and energy balance in male and female mice. Neuroscience.

[141] Zakariassen HL (2020). Central control of energy balance by amylin and calcitonin receptor agonists and their potential for treatment of metabolic diseases. Basic Clin Pharmacol Toxicol.

[142] Gedulin BR (2006). Role of endogenous amylin in glucagon secretion and gastric emptying in rats demonstrated with the selective antagonist, AC187. Regul Pept.

[143] Vella A (2002). Effects of pramlintide, an amylin analogue, on gastric emptying in type 1 and 2 diabetes mellitus. Neurogastroenterol Motil.

[144] Frias JP (2023). Efficacy and safety of co-administered once-weekly cagrilintide 2·4 mg with once-weekly semaglutide 2·4 mg in type 2 diabetes: a multicentre, randomised, double-blind, active-controlled, phase 2 trial. Lancet.

[145] Singh-Franco D (2011). The effect of pramlintide acetate on glycemic control and weight in patients with type 2 diabetes mellitus and in obese patients without diabetes: a systematic review and meta-analysis. Diabetes Obes Metab.

[146] Foll CL, Lutz TA (2020). Systemic and central amylin, amylin receptor signaling, and their physiological and pathophysiological roles in metabolism. Compr Physiol.

[147] Copp DH, Cheney B (1962). Calcitonin-a hormone from the parathyroid which lowers the calcium-level of the blood. Nature.

[148] Guida C (2019). PYY plays a key role in the resolution of diabetes following bariatric surgery in humans. EBioMedicine.

[149] Ramracheya RD (2016). PYY-dependent restoration of impaired insulin and glucagon secretion in type 2 diabetes following Roux-En-Y gastric bypass surgery. Cell Rep.

[150] Allen JM (1984). Effects of peptide YY and neuropeptide Y on gastric emptying in man. Digestion.

[151] Savage AP (1987). Effects of peptide YY (PYY) on mouth to caecum intestinal transit time and on the rate of gastric emptying in healthy volunteers. Gut.

[152] Tan TM (2021). Safety and efficacy of an extended-release peptide YY analogue for obesity: A randomized, placebo-controlled, phase 1 trial. Diabetes Obes Metab.

