# Supplementary material for: The science of safety: adverse effects of GLP-1 receptor agonists as glucose-lowering and obesity medications
Source: J Clin Invest. 2026 Feb 16;136(4):e194740. doi: 10.1172/JCI194740 (PMC12904723; doi:10.1172/JCI194740)
Supplement: Supplemental data [file jci-136-194740-s030.pdf]

Online Supplementary Material regarding the review manuscript

# The science of safety: Adverse effects of GLP-1 based glucose-lowering and obesity medications

Ryan J. Jalleh[1,2], ORCID 0000-0003-4969-4771, ryan.jalleh@adelaide.edu.au

Nicholas J. Talley[3], ORCID 0000-0003-2537-3092, nicholas.talley@newcastle.edu.au

Michael Horowitz[1,2], ORCID 0000-0002-0942-0306, michael.horowitz@adelaide.edu.au

Michael A. Nauck[4,5], ORCID 0000-0002-5749-6954, michael.nauck@rub.de

[1]Endocrine and Metabolic Unit, Royal Adelaide Hospital, Adelaide, Australia

[2]Adelaide Medical School, The University of Adelaide, Adelaide, Australia

[3]School of Medicine and Public Health, College of Health, Medicine and Wellbeing, University of Newcastle, Callaghan, NSW 2308, Australia

[4]Diabetes, Endocrinology, Metabolism Section, Medical Department I, Katholisches Klinikum Bochum gGmbH, Sankt Josef-Hospital, Ruhr-University, Bochum, Germany

[5]Institute for Clinical Chemistry and Laboratory Medicine, University Medicine Greifswald, Greifswald, Germany

**Supplemental Table 1.** A score summarizing gastrointestinal symptoms and their potential role in discontinuing treatment because of adverse events for people with type 2 diabetes or clinical obesity treated with GLP-1 receptor agonists, the dual GIP/GLP-1 receptor co-agonist tirzepatide, and next-generation incretin-based medications.

| | Severity determining score | | | | Discontinuation of treatment due to adverse events |
|---|---|---|---|---|---|
| Grade | Non-disturbing | mild | intermediate | severe | |
| | Score | | | | |
| Score | 0 | 1 | 2 | 3 | 5 |
| Symptom | | | | | |
| Gastro-esophageal acid reflux | • Occasional symptoms, rapid and spontaneous recovery | • Occasional symptoms, sometimes prolonged episodes, spontaneous recovery | • Repeated symptoms occasionally needing symptomatic treatment | • Persistent symptoms needing continuous proton pump inhibitor | unlikely |
| Nausea | • Occasional mild, short-lasting symptoms, immediate and spontaneous recovery | • Occasional symptoms, sometimes prolonged episodes, spontaneous recovery | • Repeated symptoms occasionally needing symptomatic treatment (anti-emetics) | • Persistent symptoms repeatedly needing treatment (anti-emetics) | expected |
| Vomiting | • No meaningful category | • Occasional regurgitation of gastric content into the oesophagus, but not into the throat or oral cavity or expelled | • More than 1 episode of vomiting, leading to rapid symptomatic relief (with or without medication) | • More than 1 episode of vomiting, accompanied by feeling ill, requiring treatment (anti-emetics) | expected |
| Diarrhea | • Minor change in stool consistency (softer) not accompanied by other symptoms | • Slightly more frequent bowel movements (≤ 2 times daily) and/or softer stool consistency, not accompanied by other symptoms | • Moderately more frequent bowel movements (≥ 2 times daily) and/or softer stool consistency, accompanied by other symptoms (e.g., urgency) | • Substantially more frequent bowel movements (≥ 3 times daily) and/or softer/watery stool consistency, accompanied by other symptoms (e.g., urgency, abdominal pain) | expected |
| Constipation | • Minor change in the frequency of bowel movements, without noticeable changes in stool consistency (harder), not accompanied by other symptoms | • Slightly reduced frequency of bowel movements (≤ 1 times daily) and/or harder stool consistency, not accompanied by other symptoms | • Moderately reduced bowel movements (≤ 1 times daily, but ≥ 3 times weekly) and/or harder stool consistency, accompanied by other symptoms (strain, abdominal discomfort or pain) | • Substantially reduced bowel movements (≤ 3 times weekly) and/or harder stool consistency, accompanied by other symptoms (strain, abdominal discomfort or pain) | expected |
| Abdominal discomfort/pain (should only be scored if not associated with any of the above symptoms) | • Occasional abdominal discomfort or pain, rapid and spontaneous recovery | • Occasional abdominal discomfort or pain, sometimes prolonged episodes, spontaneous recovery | • Repeated symptoms occasionally needing symptomatic treatment (e.g., anti-cholinergic medications) | • Persistent symptoms repeatedly needing treatment (e.g., anti-cholinergic medications) | unlikely |

Supplemental Table 2. Studies assessing the risk for residual gastric content (RGC; a) and aspiration (A; b) in the settings "upper gastrointestinal endoscopy" (UGE) and "general anaesthesia/surgery" (GA/S) in association with GLP-1RA therapy

| Setting | Reference | Participants (n) | | % DM | | N (%) with RGC | | Difference | |
|---|---|---|---|---|---|---|---|---|---|
| (a) RGC | First author, year | GLP-1 RA + | GLP-1 RA - | GLP-1 RA + | GLP-1 RA - | GLP-1 RA + | GLP-1 RA - | RR, OR, PR | Commentary |
| UGE | Silveira 2023 (1) | 33 | 371 | n.a. | n.a. | 8 (24.2) | 19 (5.1) | PR 5.2 | Risk ↑ with diabetes, CKD, cerbrovascular disease |
| UGE | Garza 2024 (2) | 306 | 306 | 87.9 | 87.6 | 39 (14.4) | 11 (4.1) | OR 4.4 | Risi ↑ only with insulin-dependent diabetes |
| UGE | Robalino Gonzaga 2024 (3) | 73 | 973 | 72.6 | 13.5 | 10 (13.7) | 15 (1.5) | OR 10.1 | Risiko ↑ with diabetes, CKD (stage 3-4) |
| UGE | Nasser 2024 (4) | 70 | 129 | 82.9 | 32.6 | 4 (8.5) | 0 (0.0) | P = 0.01 | Risk ↓ with same-day colonoscopy |
| UGE | Nadeem 2024 (5) | 922 | 34261 | 82.0 | 15.5 | 125 (13.6) | 788 (2.3) | OR 4.1 | Risk ↑ for discontinuation of endoscopy/scheduling of another attempt |
| UGE | Wu 2024 (6) | 90 | 102 | 68.9 | 24.5 | 19 (18.6) | 5 (5.6) | OR 4.8 | - |
| UGE | Chapman 2024 (7) | 84 | 84 | 86.9 | 84.5 | 11 (13.1) | 4 (4.8) | aOR 4.6 | - |
| UGE | Quinn 2025 (8) | 470 | 470 | 74.5 | 20.6 | 59 (12.6) | 26 (5.5) | OR 1.0 | - |
| GA/S | Sherwin 2023 (9) | 10 | 10 | 10.0 | 0.0 | 9 (90.0) | 1 (10.0) | P = 0.005 | Diagnosis with ultrasound in lateral position |
| GA/S | Nersessian 2024 (10) | 107 | 113 | 0.0 | 0,0 | 43 (40.2) | 3 (2.7) | OR 24.6 | No reduction in risk with durations between 1 and 10 days since last administration of long-acting GLP-1 RA |
| GA/S | Sen 2024 (11) | 62 | 62 | 71.0 | 24,2 | 35 (56.5) | 12 (19.4) | PR 2.9 | No reduction in risk with durations between 1 and 17 days since last administration of long-acting GLP-1 RA |
| All | | 2227 | 36881 | 74.8 | 16,4 | 362 (16.3) | 884 (2.4) | Risk ↑ with GLP-1RAs | Risk ↑ by approximately 7-fold |
| | | | | | | N (%) mit aspiration/pneumonia | | Difference | |
| (b) Aspiration | | GLP-1 RA + | GLP-1 RA - | GLP-1 RA + | GLP-1 RA - | GLP-1 RA + | GLP-1 RA - | RR, OR, PR | Commentary |
| UGE | Peng 2024 (12) | 29320 | 52184 | 78.4 | 69,2 | 160 (0.5) | 143 (0.3) | RR 1.1 | - |
| UGE | Yeo 2024 (13) | 15908 | 15908 | 71.5 | 72,7 | 132 (0.8) | 100 (0.6) | HR 1.3, p = 0,036 | Risk ↓ with same-day colonoscopy |
| UGE | Alkabbani 2024 (14) | 24317 | 18537 | 100.0 | 100,0 | 103 (0.4) | 79 (0.4) | RR 1.0 | Comparison to SGLT-2 inhibitor therapy |
| UGE | Velji-Ibrahim 2025 (15) | 11477 | 11477 | 76.1 | 78,7 | 20 (0.1) | 22 (0.1) | HR 0.9 | - |
| GA/S | Welk 2024 (16) | 3833 | 14072 | 100.1 | 100,1 | 10 (0.3) | 49 (0.3) | RR 0.7 | General and spinal anaesthesia without difference |
| GA/S | Klonoff 2024 (17) | 2256 | 11405 | 98.4 | 97,8 | 9 (0.4) | 70 (0.6) | OR 0.8 | - |
| GA/S | Buddhiraju 2024 (18) a (THA) | 1044 | 1044 | 69.3 | 68,3 | 0 (0.0) | 0 (0.0) | - | No difference regarding other post-anaesthesia/post-surgery complications |
| GA/S | Buddhiraju 2024 (18) b (TKA) | 2095 | 2095 | 68.9 | 68,4 | 10 (0.5) | 10 (0.5) | RR 1.0 | No difference regarding other post-anaesthesia/post-surgery complications |
| GA/S | Chen 2025 (19) | 5931 | 360545 | 84.3 | 17,1 | 42 (0.7) | 2335 (0.6) | HR 0.8 | Risk ↑ with higher age, diabetes, obesity, CKD, and cerebrovascular disease |
| GA/S | Poeran 2025 (20) | 17665 | 17665 | 13.2 | 13,2 | 14 (0.1) | 12 (0.1) | OR 0.8 | - |
| GA/S | Wright 2025 (21) | 31488 | 31488 | 68.3 | 69,0 | 252 (0.8) | 110 (0.7) | OR 1.1 | - |
| All | | 145334 | 536420 | 65.1 | 35,1 | 752 (0.5) | 2930 (0.5) | Risk unchanged | Overall low risk (approximately 0.5 %) |

A: Aspiration; GA/S: general anaesthesia/surgery; aOR: adjusted odds ratio; DM: Diabetes mellitus; UGE: Endoscopy of the upper gastrointestinal tract; GLP-1 RAs: GLP-1 receptor agonists; HR: Hazard ratio; OR: Odds ratio; PR: Prevalence ratio; PSM: Propensity score matching; RGC: Residual gastric content; RR: Relative risk: T2D: Type 2 Diabetes; THA: Total hip arthroplasty; TKA: total knee arthroplasty

## References


1. Silveira SQ, da Silva LM, de Campos Vieira Abib A, de Moura DTH, de Moura EGH, Santos LB, et al. Relationship between perioperative semaglutide use and residual gastric content: A retrospective analysis of patients undergoing elective upper endoscopy. *J Clin Anesth.* 2023;87:111091.
2. Garza K, Aminpour E, Shah J, Mehta B, Early D, Gyawali CP, et al. Glucagon-like peptide-1 receptor agonists increase solid gastric residue rates on upper endoscopy especially in patients with complicated diabetes: A case-control study. *Am J Gastroenterol.* 2024;119:1081-8.
3. Robalino Gonzaga E, Farooq A, Mohammed A, Chandan S, Fawwaz B, Singh G, et al. Real-world impact of GLP-1 receptor agonists on endoscopic patient outcomes in an ambulatory setting: A retrospective study at a large tertiary center. *J Clin Med.* 2024;13:Published online 12 September 2024.
4. Nasser J, Hosseini A, Barlow G, Gianchandani R, Rezaie A, Pimentel M, et al. Food retention at endoscopy among adults using glucagon-like peptide-1 receptor agonists. *JAMA Netw Open.* 2024;7:e2436783.
5. Nadeem D, Taye M, Still MD, McShea S, Satterfield D, Dove JT, et al. Effects of glucagon-like peptide-1 receptor agonists on upper endoscopy in diabetic and nondiabetic patients. *Gastrointest Endosc.* 2024;100:745-9.
6. Wu F, Smith MR, Mueller AL, Klapman SA, Everett LL, Houle T, et al. Association of glucagon-like peptide receptor 1 agonist therapy with the presence of gastric contents in fasting patients undergoing endoscopy under anesthesia care: a historical cohort study. *Can J Anaesth.* 2024;71:958-66.
7. Chapman MB, Norwood DA, Price C, Abdulhadi B, Kyanam Kabir Baig K, Ahmed AM, et al. Effects of glucagon-like peptide-1 receptor agonists on gastric mucosal visibility and retained gastric contents during EGD. *Gastrointest Endosc.* 2024;100:923-7.
8. Quinn JA, Welch KM, Fujino E, Jimenez Rosado CA, An X, Schoenherr JW, et al. Perioperative glucagon-like peptide-1 receptor agonist use and retained gastric contents: A retrospective analysis of patients undergoing elective upper endoscopy. *J Clin Anesth.* 2025;102:111776.
9. Sherwin M, Hamburger J, Katz D, and DeMaria S, Jr. Influence of semaglutide use on the presence of residual gastric solids on gastric ultrasound: a prospective observational study in volunteers without obesity recently started on semaglutide. *Can J Anaesth.* 2023;70:1300-6.
10. Nersessian RSF, da Silva LM, Carvalho MAS, Silveira SQ, Abib ACV, Bellicieri FN, et al. Relationship between residual gastric content and peri-operative semaglutide use assessed by gastric ultrasound: a prospective observational study. *Anaesthesia.* 2024;79:1317-24.
11. Sen S, Potnuru PP, Hernandez N, Goehl C, Praestholm C, Sridhar S, et al. Glucagon-like peptide-1 receptor agonist use and residual gastric content before anesthesia. *JAMA Surg.* 2024.
12. Peng CY, Chang YC, Gong C, Chang Y, Chi KY, Hsiao CL, et al. Association between glucagon-like peptide-1 receptor agonists and aspiration pneumonia during endoscopic procedures. *Anesthesiology.* 2024;141:1009-12.
13. Yeo YH, Gaddam S, Ng WH, Huang PC, Motility, Metabolic Pharmacoepidemiology G, et al. Increased risk of aspiration pneumonia associated with endoscopic procedures among patients with glucagon-like peptide 1 receptor agonist use. *Gastroenterology.* 2024.
14. Alkabbani W, Suissa K, Gu KD, Cromer SJ, Paik JM, Bykov K, et al. Glucagon-like peptide-1 receptor agonists before upper gastrointestinal endoscopy and risk of

pulmonary aspiration or discontinuation of procedure: cohort study. *BMJ.* 2024;387:e080340.

15. Velji-Ibrahim J, Nathani P, Patel HK, and Sharma P. GLP-1 receptor agonist use does not increase risk of respiratory complications post-endoscopy. *Endosc Int Open.* 2025;13:a24872937.
16. Welk B, McClure JA, Carter B, Clarke C, Dubois L, and Clemens KK. No association between semaglutide and postoperative pneumonia in people with diabetes undergoing elective surgery. *Diabetes Obes Metab.* 2024;26:4105-10.
17. Klonoff DC, Kim SH, Galindo RJ, Joseph JI, Garrett V, Gombar S, et al. Risks of peri- and postoperative complications with glucagon-like peptide-1 receptor agonists. *Diabetes Obes Metab.* 2024;26:3128-36.
18. Buddhiraju A, Kagabo W, Khanuja HS, Oni JK, Nikkel LE, and Hegde V. Decreased risk of readmission and complications with preoperative GLP-1 analog use in patients undergoing primary total joint arthroplasty. *J Arthroplasty.* 2024;39:2911-5 e1.
19. Chen YH, Zink T, Chen YW, Nin DZ, Talmo CT, Hollenbeck BL, et al. Postoperative Aspiration Pneumonia Among Adults Using GLP-1 Receptor Agonists. *JAMA Netw Open.* 2025;8(3):e250081.
20. Poeran J, Iban YC, Zhong H, Illescas A, Cozowicz C, Reisinger L, et al. Preoperative GLP-1 agonist use is not associated with perioperative aspiration or pneumonia: an observational study using US national data. *Br J Anaesth.* 2025;134:1526-8.
21. Wright JD, Chen L, Xu X, Hur C, Matsuo K, Elkin EB, et al. Glucagon-like-peptide-1 (GLP-1) receptor agonist use and the risk of pulmonary aspiration in patients undergoing surgery. *Int J Surg.* 2025;111:4090-3.
